# Cell-derived Nanoparticles Provide a Robust Platform to Manufacture Therapeutic T cells

**DOI:** 10.21203/rs.3.rs-8436008/v1

**Published:** 2026-02-04

**Authors:** Mosha Z. Deng, Tanishk Sinha, Meizan Lai, Shuguang Jiang, Hong Kong, Vincent Cooper, Meidi Gu, Jonah Perelman, Caimei Zhang, Chiquita Hanindya, Victor Carpio, Peter Keller, Julie K. Jadlowsky, Rachel Leskowitz, Stephen McKenna, Megan Four, Jesus Arturo Junco Barranco, Natalie Cooper, Ankita Jain, Kathleen Haines, Mercy Gohil, Laura Garcia-Gerique, Athena Russell, Irina Kulikovskaya, Vanessa Gonzalez, Joseph A. Fraietta, Gabriela Plesa, Neil C. Sheppard, Megan M. Davis, Anne Chew, Elizabeth Hexner, Noelle Frey, Carl H. June, Jakub Svoboda, James L. Riley

**Affiliations:** a Department of Microbiology, University of Pennsylvania, Philadelphia PA 19104 USA.; b Center for Cellular Immunotherapies, University of Pennsylvania, Philadelphia PA 19104 USA.; c BlueWhale Bio, Philadelphia PA 19104 USA.; d Department of Pathology and Laboratory Medicine, University of Pennsylvania, Philadelphia PA.; e Abramson Cancer Center, University of Pennsylvania, Philadelphia PA.; f Parker Institute for Cancer Immunotherapy, Perelman School of Medicine University of Pennsylvania, Philadelphia, PA 19104 USA.

## Abstract

Some patients cannot receive T cell therapies because their cells are unable to be manufactured. To address this limitation, we developed K562-based artificial antigen-presenting cells (aAPCs) expressing an OKT3-derived scFv for TCR stimulation, CD86 and 4–1BBL for costimulation, and membrane-bound IL-7 and IL-15Rα/IL-15 for cytokine support. From these aAPCs, we generated cell-derived nanoparticles (CDNPs) that accelerated T cell entry into the cell cycle compared with CD3/28-coated beads, enabling efficient concurrent activation and lentiviral transduction. CDNPs robustly expanded T cells from patients whose products could not be manufactured using standard approaches, and these CDNP-derived CAR T cells controlled tumors in humanized mouse models. In a phase I trial of patients with CD19^+^ malignancies (NCT04684563), cGMP-compatible CDNPs enabled streamlined 3-day manufacturing of IL-18–expressing CD19 CAR T cells, yielding higher cell recovery and durable clinical responses without unexpected toxicities, supporting CDNPs as a platform for commercial CAR T cell production.

Adoptively transferred T cells have revolutionized the way some cancers and autoimmune diseases are treated, resulting in markedly better outcomes for patients^1–4^. Many factors influence the extent by which a patient benefits from this therapy including the initial CD4:CD8 ratio in the apheresis product^5–7^, the integration site of vector within the T cell genome^8,9^, relative proportion of memory T cell subsets in the starting apheresis material^10–12^, and the previous therapies administered prior to T cell therapy^13,14^. Recently, a correlation between how well T cells expand during *ex vivo* manufacturing and improved clinical outcomes was noted for individuals treated with a CD19-specific CARTs^12,15^. These studies suggest improved *ex vivo* T cell manufacturing may result in better clinical outcomes. Additionally, a subset of individuals (5–30%) are unable to receive this lifesaving therapy because their T cells are refractory to *ex vivo* expansion using state-of-the-art approaches or their disease progresses during the vein-to-vein autologous CART cell interval, an issue that can be mitigated by rapid manufacturing^16–30^. Thus, T cell manufacturing platforms that can generate highly functional engineered T cells from a greater number of patients with reduced manufacturing times are needed. Lastly, CART manufacturing is a personalized therapy that is both expensive and complicated to implement on a commerical scale^31,32^. Therefore, improvements that streamline and improve T cell manufacturing will result in more effective and affordable therapies that are able to benefit more people.

Paramagnetic beads coated with CD3 and CD28 specific antibodies (CD3/28 beads) enable the *ex vivo* expansion of T cells that durably engraft upon re-infusion^33–36^. While these magnetic beads were instrumental in the pivotal studies that highlighted the therapeutic potential of CARTs and are used to generate commercial CARTs^37^, they have considerable drawbacks: 1) only two signals are used to activate T cells; 2) the use of antibodies rather than natural ligands to trigger activating T cell receptors, which may alter the signals T cells receive^38–40^; and 3) the need to remove these beads prior to infusion, which is labor intensive and results in the substantial loss of CARTs, limiting rapid T cell manufacturing strategies due to their lower yield. Other platforms are now available that do not need to be removed prior to T cell infusion^41–43^, but these still suffer from using Abs to engage only CD3 and CD28. To overcome these shortcomings, cell-based artificial APCs (aAPCs) were generated^44–46^ that readily allowed the addition of multiple natural costimulatory and cytokine stimuli^47^, and these dramatically improved the expansion of difficult to propagate T cells such as tumor infiltrating lymphocytes (TILs)^48^ and regulatory T cells (Tregs)^49–51^. While K562-based aAPCs produced through current good manufacturing practices (cGMP) proved effective to manufacture T cells for adoptive T cell therapy clinical trials^52,53^ (NCT01369875, NCT00512889, NCT01555892, NCT01362452), concerns about using an irradiated tumor cell for T cell manufacturing have limited their widespread use. To alleviate this concern, we have generated cell-derived nanoparticles (CDNPs) through a nitrogen cavitation process^54^. We investigated these ready-to-use complexes in pre-clinical *in vitro* and *in vivo* models and ultimately produced cGMP-compatible CDNPs which were used to generated CD19-specific CARTs armored to express IL-18 which were administered to patients with relapsed/refractory hematologic malignancies as part of the phase I study (NCT04684563).

## Results

### Construction of a “ready-to-use” aAPC.

**Figure 1:**
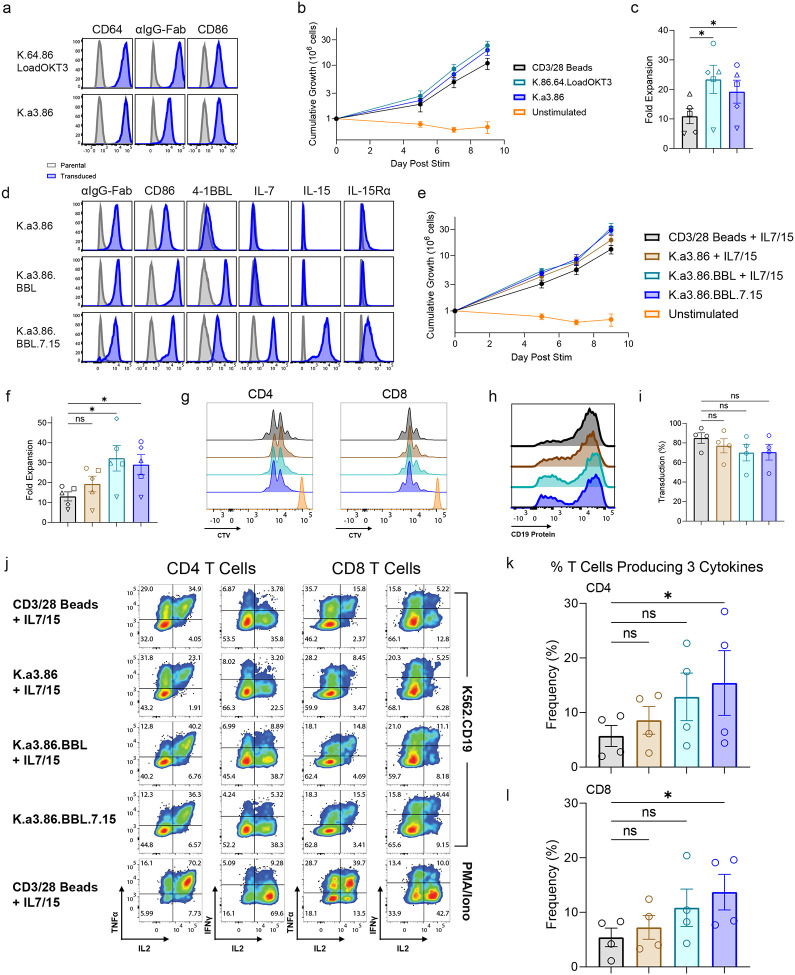
Construction of a “ready-to-use” aAPC. **a**, K562 cells were transduced with an anti-CD3 scFv and CD86 and single cell sorted to generate K.a3.86 aAPCs. The surface expression of anti-CD3 scFv on these aAPCs and previously described K.64.86 aAPC loaded with OKT3 mAb and anti-CD86 Ab was evaluated by flow cytometry; both anti-CD3 scFv and OKT3 bound by CD64 was detected with a Goat antibody against mouse IgG F(ab’)2. **b**,**c** Primary human T cells were activated with the indicated aAPC, transduced with 19BBz CAR one day later, and counted after 5, 7, and 9 days of culture (**b**), Cumulative fold expansion was calculated in (**c**) (n=5). **d**, The indicated genes were introduced via lentiviral transduction to generate the indicated aAPCs. **e**,**f**, T cells were activated by the indicated aAPCs, transduced with 19BBz and counted and re-fed after 5, 7, and 9 days of culture (**e**); cumulative fold expansion is shown in **f** (n=5). **g**, CD4 and CD8 T cells were labeled with CellTraceViolet (CTV), activated by the indicated aAPC, and CTV dilution was measured by flow cytometry. **h**,**i**, T cells were activated with the indicated aAPC, transduced with 19BBz, and transduction efficiency was measured 7 days later for a representative experiment (**h**) or summarized for 4 independent experiments (**i**); surface CAR was detected using soluble CD19 protein. **j-l**, T cells from **e-i** were mixed with K562 cells expressing CD19 at 1:2 E:T ratio for 4h and intracellular IL-2, TNF, and IFNγ were measured by flow cytometry. **j** shows one representative experiment and the number of T cells producing all three cytokines was determined for CD4 (**k**) and CD8 (**l**) T cells. All data are from at least three independent donors and expressed as mean ± SEM. **c**,**f**, Repeated measures one-way analysis of variance (ANOVA) with Holm-Sidak’s post hoc test was used for pairwise, log-transformed comparisons among three or more groups of one factor. **i**, Repeated measures one-way ANOVA with Tukey’s post hoc test was used for pairwise comparisons among three or more groups of one factor. **k**,**l**, Friedman’s test followed by Dunn’s multiple comparisons test was used for nonparametric pairwise comparisons among three or more groups of one factor. The same color schemes apply to figures **a** and **d**, the same color schemes apply to figures **b** and **c**, and the same color schemes apply to figures **e**-**i**, **k** and **l**.

Our previous K562-based aAPCs expressed CD64 (K.64.86), which could bind an anti-CD3 mAb (OKT3), and deliver signal one to T cells^44,49^. However, this loading proved cumbersome to standardize for use in cGMP manufacturing. As an alternative, we transduced a molecule comprised of a CD3-specific scFv linked to a transmembrane domain and a short non-signaling cytoplasmic tail into K562 cells expressing CD86 and compared these “ready-to-use” aAPCs with OKT3 mAb-loaded K.64.86 aAPCs in their ability to expand primary human T cells. While the OKT3 scFv-transduced aAPCs had less anti-CD3 Ab on its cell surface compared to CD64 expressing aAPCs loaded with OKT3 mAb (Fig. 1a), we observed similar levels of expansion of T cells after 9 days of culture using either of these aAPCs, and both of these approaches were superior to the number of T cells expanded when CD3/28 beads were used to activate the T cells (Fig. 1b,c). The ability to add additional costimulatory and/or cytokine signals to aAPCs allows for the preferential expansion of T cell subsets^55^ and enhances T cell activation, which results in better lentiviral transduction and *ex vivo* expansion^56,57^. Given the widespread use and success of CD19-specific CART therapy, we designed an aAPC to manufacture these cells based on the current literature. In addition to using CD86, the natural ligand for CD28 and the most potent costimulatory molecule^58^, we also added 4–1BBL, which provides important survival and activation signals to T cells and particularly CD8 T cells^45^. Furthermore, the addition of IL-7 and IL-15 has been shown to preserve less differentiated, stem cell-like memory T cells during *ex vivo* manufacturing^59,60^. Thus, we genetically engineered K562 cells to produce IL-7, which was linked to mCherry via a T2A sequence, IL-15, and IL-15Rα (CD215). Similar to what occurs naturally *in vivo*, we observed IL-15 binding to IL-15Rα, which allows the cytokine to be “transpresented” to T cells^61^ (Fig. 1d). Compared to T cells activated by CD3/28 beads, all aAPCs expanded T cells to a greater extent. (Fig. 1e,f). Interestingly, we found that the capacity to induce cell division was similar between activation substrates, suggesting that differences in the total number of T cells expanded resulted from enhanced proliferation and/or survival after the initial stimuli (Fig. 1g).

To assess the function of T cells expanded by the various aAPCs, we transduced T cells with a CD19-specific CAR fused to the 4–1BB signaling domain (19BBz)^62^. We found similar transduction efficiency of the CD19 CAR regardless of activation substrate (Fig. 1h,i). Anti-tumor effector function, as measured by intracellular cytokine staining after stimulation by tumor cells expressing CD19 was comparable overall between different activation substrate groups. Interestingly, CARTs activated with the aAPC presenting the full complement of anti-CD3 scFv, CD86, 4–1BBL, IL-7, and IL-15 signals (hereafter referred to as “K.a3.86.BBL.7.15”) maintained a significantly higher proportion of highly polyfunctional CARTs (producing at least three cytokines) in both CD4 and CD8 T cells (Fig. 1j–l). Together, this data shows that K.a3.86.BBL.7.15 aAPC robustly expanded CARTs.

### Cavitation of aAPCs generates CDNPs that retain costimulatory and cytokine activity and enable simultaneous transduction and activation of CARTs.

**Figure 2:**
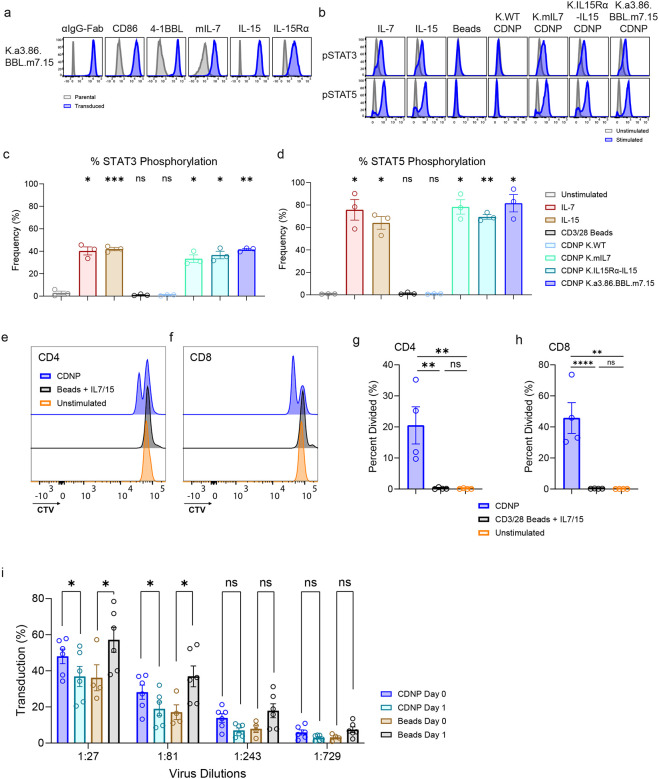
Cavitation of aAPCs generates CDNPs that retain costimulatory and cytokine activity and enable simultaneous transduction and activation of CARTs. **a**, Phenotype of K.a3.86.BBL.m7.15 aAPC determined by flow cytometry **b**, Freshly isolated human T cells were stimulated for 20 minutes with the indicated CDNP, CD3/28 coated beads, 10 ng/mL of IL-7 or 10 ng/mL of IL-15 and intracellular pSTAT3 and pSTAT5 were measured by flow cytometry. **c,d**, Summary STAT3 and STAT5 phosphorylation of T cells from **b** (n=3). **e-h**, Primary human CD4 (**e,g**) and CD8 (**f,h**) T cells were labeled with CTV and activated with the indicated aAPCs for 2 days. Summary data for 4 independent experiments is shown in **g,h**. **i**, T cells activated by the indicated aAPC were transduced immediately (Day 0) or the following day (Day 1) with the indicated dilution of lentiviral vectors carrying various transgenes, including 19BBz CAR, GFP, and mCherry. All data are from at least three independent experiments using unique donors and expressed as mean ± SEM. **c**,**d**, Repeated measures one-way ANOVA with Dunnett’s post hoc test was used for pairwise comparison of three or more groups of one factor; statistical significance was calculated via comparison with the unstimulated sample. **g**,**h**, Repeated measures one-way ANOVA with Tukey’s post hoc test was used for pairwise, log-transformed comparisons among three or more groups of one factor. **i**, Mixed effects one-way ANOVA with Sidak’s multiple comparisons test.

Next, we sought to investigate whether CDNPs from these aAPCs could retain the benefits of aAPCs to expand T cells without the risk of using an intact, albeit irradiated, tumor cell line to manufacture T cells. Previous work showed that nitrogen cavitation of K562 aAPCs expressing a membrane bound version of IL-21 could expand NK cells and that this process could be adapted for clinical use^54,63^ (NCT04395092). We reasoned that we would need to generate a membrane-bound version of IL-7 to retain this activity in the purified CDNP product. Moreover, we were unsure whether IL-15 bound to IL-15Rα (“IL-15Rα/IL-15”) complex would be retained after the CDNP purification process. To determine whether membrane-bound IL-7 had similar activity to soluble IL-7 and whether IL-15 signaling remained intact in CDNPs, we first re-engineered the K.a3.86.BBL.7.15 aAPC by replacing IL-7 with a membrane bound version of IL-7 (“mIL-7”) to generate K.a3.86.BBL.m7.15 (Fig 2a). We also generated K562 cell lines that only expressed mIL-7 or IL-15Rα/IL-15 (Supplementary Fig. 2) so we could study the ability of CDNP-associated cytokines to signal in isolation. CDNPs were generated from K562 parental cells (“K.WT”), K.mIL-7, K.IL-15Rα/IL-15, and K.a3.86.BBL.m7.15 aAPCs by subjecting them to 2 rounds of cavitation followed by a purification and concentration process. We then compared the ability of soluble cytokines and each of these CDNPs to activate STAT3 and STAT5 when mixed with freshly isolated human T cells^64–67^. Neither K.WT CDNPs or CD3/28 beads could activate STAT3 or STAT5, but all the CDNPs that presented mIL-7 or IL-15Rα/IL-15 alone or in combination could robustly activate STAT3 and STAT5, demonstrating that cytokines attached to the CDNPs were functional (Fig. 2b–d).

We next determined the optimal ratio of K.a3.86.BBL.m7.15 CDNPs (now referred to as simply CDNPs) to T cells and compared the growth kinetics of T cells expanded by CDNPs with those activated by CD3/28 beads + IL-7 and IL-15. Notably, CDNPs led to significantly faster cell division in both CD4 and CD8 T cells where ~20% of CD4 T cells and ~40% of CD8 T cells divided once after two days of stimulation, whereas a negligible percentage of T cells divided after activation by CD3/28 beads + IL-7 and IL-15 (Fig. 2e–h). This proliferation advantage in CDNP-activated T cells continued to be observed three and four days after activation (Supplementary Fig. 1).

The rapid division of CDNP-activated T cells suggested that this activation substrate may allow for simultaneous transduction and activation (hereafter referred to as Day 0 transduction), which would simplify the T cell manufacturing process. We found that Day 0 transduction was not only feasible in CDNP-activated T cells but also resulted in higher levels of transduction relative to T cells transduced after 1 day of CDNP stimulation (Fig. 2i). Using a wide range of vector concentrations, we observed that Day 0 transduced, CDNP-activated T cells outperformed Day 1 transduced, CDNP-activated T cells, whereas the reverse was true for CD3/28 bead-stimulated T cells. Subsequently, we employed Day 0 transduction in the manufacturing of all CDNP-activated CARTs. These data show that CDNPs retain all the beneficial aspects of cell-based aAPCs and offer additional benefits such as the ability to perform simultaneous transduction and activation.

### CDNP stimulation leads to functional CART generation.

**Figure 3:**
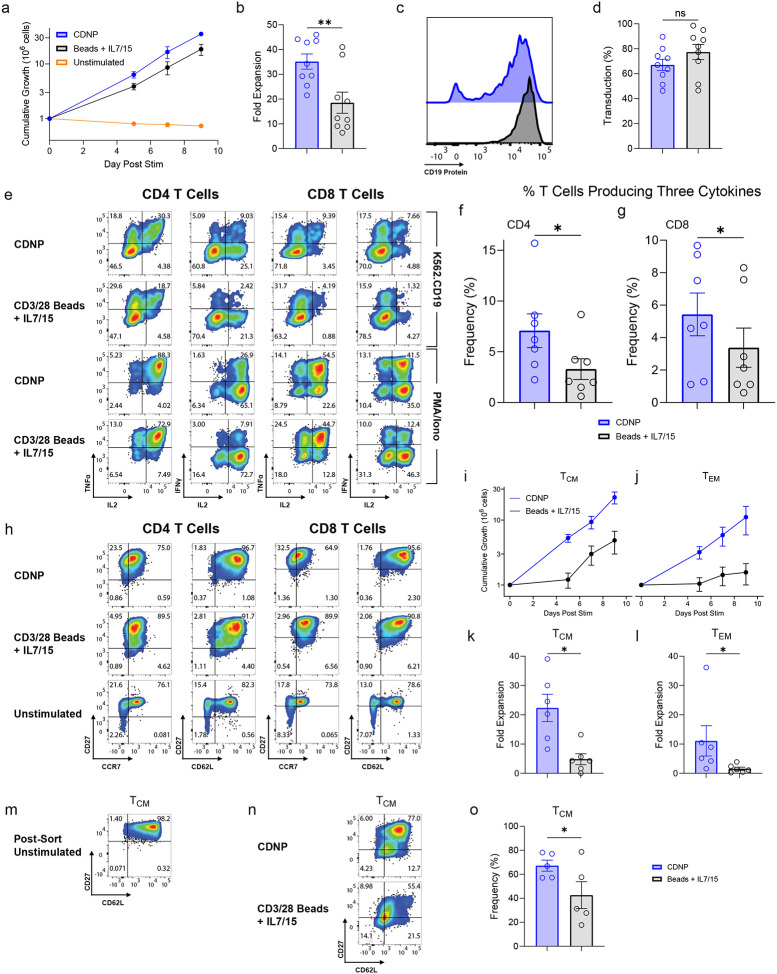
CDNP stimulation leads to functional CART generation. **a-h,** T cells were activated by CDNPs or CD3/28 beads + IL-7 and IL-15 and transduced with 19BBz CAR on Day 0 (CDNP) or Day 1 (Beads), and counted and re-fed after 5, 7, and 9 days of culture (**a**), (n=9). **b**, Cumulative fold expansion was measured (n=9). Transduction efficiency was assessed using labeled soluble CD19 protein 7 days later for a representative experiment (**c**) or summarized for 9 independent experiments (**d**); **e**, T cells from **a**-**d** were mixed with K562 cells expressing CD19 at 1:2 E:T ratio for 4h and intracellular IL-2, TNF, and IFNγ were measured by flow cytometry. **f**,**g**, The number of T cells producing all three cytokines was determined for CD4 (**f**) and CD8 (**g**) T cells (n=7). **h**, T cells from **a-d** were stained with CD45RA, CD27, CD62-L, and CCR7 antibodies after 7 days of culture. Data is representative of at least three independent experiments. **i**-**n** CD45RA-CD27+CCR7+ (**i**,**k,m,n**) and CD45RA-CD27−CCR7− (**j**,**l**) T cells were sorted and activated by CD3/28 beads + IL-7 and IL-15 or CDNPs and counted after 5, 7 and 9 days of culture. Fold expansion after 9 days of culture was calculated (**k**,**l**), (n=6). Freshly sorted CD45RA-CD27+CCR7+ T cells (**m**) were expanded for at least 7 days (**n**). (**o**) Summarized data for 5 independent experiments is shown. All data are from at least three independent experiments using unique donors and expressed as mean ± SEM. **b**,**k**,**l,o**, Two-sided ratio t test was used in pairwise, log-normal comparisons between two groups. **d**, Two-sided t test was used in pairwise comparisons between two groups. **f**,**g**, Two-sided, Wilcoxon matched-pairs signed-rank test was used in nonparametric pairwise comparison of two groups. The same color schemes apply to figures **a**-**d**, **f**, **g**, **i**-**l**, and **o**.

Next, we wanted to determine how well CDNPs manufacture CARTs. We observed that CARTs stimulated with CDNPs underwent superior expansion compared to those activated by CD3/28 beads + IL-7 and IL-15 (Fig. 3a,b). CAR transduction efficiency remained comparable between different activation substrate groups (Fig. 3c,d). Similar to what was observed with cell-based aAPCs, CDNP-activated CARTs maintained a significantly higher proportion of highly polyfunctional CARTs (producing at least three cytokines) in both CD4 and CD8 T cells when co-cultured with CD19+ tumor cells (Fig 3e–g). We also examined the relative proportion of memory subsets of CARTs present before and after expansion with CDNPs or CD3/28 beads + IL-7 and IL-15. T cells can be classified into a large number of subsets based on the expression of costimulatory, trafficking and chemokine receptors such as CD27, CD62L, and CCR7, and the relative ratio of these subsets can differ substantially between healthy individuals and patients^11,68^. We found that activation with either CDNPs or CD3/28 beads + IL-7 and IL-15 led to a similar enrichment of central memory T cells (defined as CD27+CCR7+ or CD27+CD62L+) (Fig. 3h). To examine how each stimulus expanded each memory population in isolation, we sorted freshly purified T cells into central memory (CD45RA-CD27+CCR7+) and effector memory (CD45RA-CD27−CCR7−) populations prior to stimulation and measured their expansion after activation with either CDNPs or CD3/28 beads + IL-7 and IL-15. While we saw a modest benefit of using CDNPs to expand central memory T cells (Fig. 3i,k), we observed more significant differences in the ability of CDNPs to expand effector memory T cells (Fig. 3j,l). Two possibilities could explain this data: either CDNPs expand effector memory cells better or CDNPs force the differentiation of central memory cells to effector memory. To distinguish between these possibilities, we sorted central memory T cells and activated them with CDNPs or CD3/28 beads + IL-7 and IL-15. We noted that CDNPs were better able to maintain the phenotype of sorted central memory subset after at least 7 days of expansion compared to CD3/28 beads + IL-7 and IL-15 (Fig. 3m–o), demonstrating the phenotypic stability of the CDNP-manufactured CART product. This suggests that the higher number of effector memory cells in cultures after CDNP activation are present because CDNPs expand this population better than CD3/28 beads + IL-7 and IL-15 rather than differentiating central memory cells to effector memory cells. Moreover, these studies suggest that CDNPs may result in improved expansion of highly differentiated patient T cells.

### CDNPs rescue expansion of patient T cells that failed the conventional CART manufacturing process.

**Figure 4:**
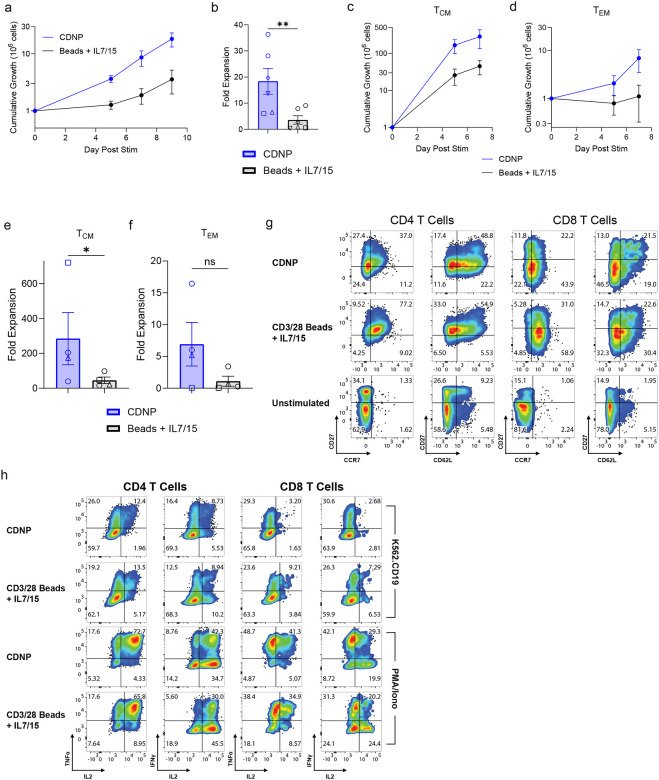
CDNPs rescue expansion of patient T cells that failed the conventional CART manufacturing process. **a**,**b**, T cells from patients whose T cells could not be manufactured using CD3/28 coated beads + IL-7 and IL-5 were activated by CDNPs or CD3/28 beads + IL-7 and IL-15, transduced with 19BBz CAR on Day 0 (CDNP) or Day 1 (Beads), counted after 5, 7, and 9 days of culture (**a**); fold expansion over 9 days of culture is shown in **b**. **c-f**, Normalized growth and fold expansion of CD27+CCR7+ central memory T cells (**c,e**) or CD27-CCR7- effector memory T cells (**d,f**) throughout the initial 7 days of culture (n=4). **g**, Representative profiling of central memory (CD27+CCR7+ or CD27+CD62L+) and effector memory (CD27−CCR7− or CD27−CD62L−) subsets of 19BBz CARTs after at least 7 days of culture. **h,** After 9 days of culture, T cells were mixed with K562 cells expressing CD19 for 4h and intracellular IL-2, TNF, and IFNγ measured by flow cytometry. All data are from at least three independent donors and expressed as mean ± SEM. **b**,**e**,**f**, Two-sided ratio t test was performed for pairwise log-normal comparisons between two groups. The same color schemes apply to figures **a**-**f**.

Given the superior ability of CDNPs to expand effector T cells compared to CD3/28 beads + IL-7 and IL-15, we sought to determine whether CDNPs could expand T cells from patients, especially those whose T cells could not be manufactured using CD3/28 beads + IL-7 and IL-15. T cells from patients with multiple myeloma (MM) and various subtypes of non-Hodgkin lymphoma (NHL), including chronic lymphocytic leukemia (CLL), mantle cell lymphoma (MCL), and follicular lymphoma (FL) whose products failed CD3/28 bead +IL-7 and IL-15 manufacturing were activated with CDNPs or CD3/28 beads + IL-7 and IL-15. Impressively, CDNPs were able to expand these previously recalcitrant T cells whereas CD3/28 beads + IL-7 and IL-15 failed to do so, confirming the previous GMP manufacturing outcome (Fig. 4a,b). Furthermore, we observed that both central and effector memory T cells expanded to greater levels when activated by CDNPs when compared to CD3/28 beads + IL-7 and IL-15 (Fig. 4c–f). The capacity of CDNPs to expand the differentiated, effector memory population is especially important here because these subsets are much more abundant in the initial starting population of patient-derived T cells compared to that of healthy donors (Fig. 3h, 4g). Furthermore, the high degree of expansion of the central memory compartment is also striking in part due to its critically low abundance in the starting apheresis of certain patients. Finally, the *in vitro* effector function of patient-derived CARTs against CD19+ tumor cells remained robust and comparable with CD3/28 bead-activated CARTs (Fig. 4h).

### CDNP-stimulated CARTs from patient T cells that failed conventional manufacturing maintain robust anti-tumor activity and prolong survival *in vivo*.

**Figure 5:**
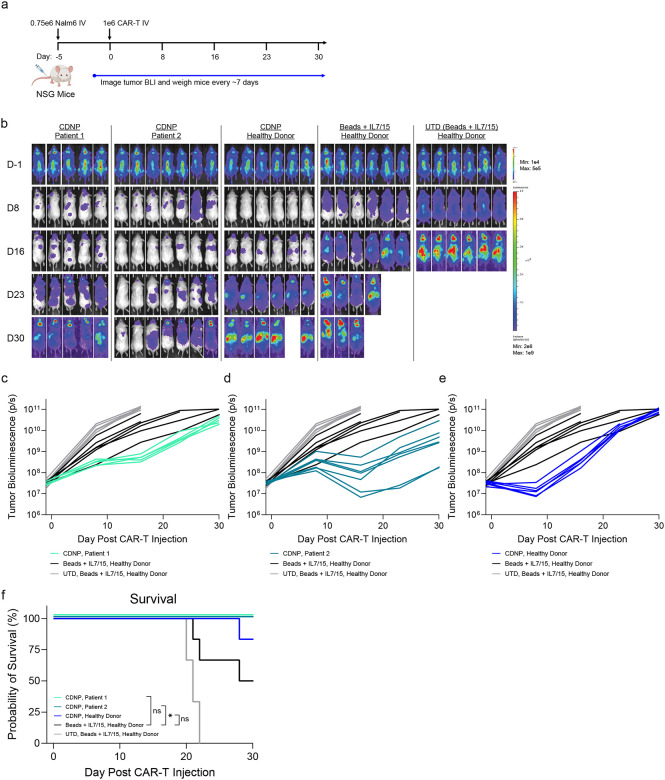
CDNP-stimulated CARTs from patient T cells that failed conventional manufacturing maintain robust anti-tumor activity and prolong survival *in vivo*. **a**, Schematic of Nalm6 xenograft model in NSG mice receiving 1 million 9-day manufactured 19BBz CARTs sourced from two patients with CLL and a healthy donor and activated by CDNPs or CD3/28 beads + IL-7 and IL-15. **b**, Tumor burden (BLI) images of mice receiving CARTs. **c-e**, Longitudinal tumor burden of NSG mice receiving 19BBz CARTs (each line represents one mouse) from CLL Patient 1 (**c**), CLL Patient 2 (**d**), or a healthy donor (**e**). **f**, Kaplan-Meier survival curve based on humane endpoints. Kaplan-Meier survival data were analyzed using a log-rank (Mantel-Cox) test. The same color schemes apply to figures **c**-**f**.

The failure to manufacture bead-activated CARTs from these patients compromised our ability to perform side-by-side *in vivo* testing of CAR-T therapy produced by CDNPs and CD3/28 beads + IL-7 and IL-15 (Fig. 4a,b; Supplementary Fig. 2). Nevertheless, we did assess the *in vivo* anti-tumor activity of CDNP manufactured products from these patient donors who were unable to received CARTs in a xenograft model of NOD/SCID/IL2Rγ−/− (NSG) mice bearing CD19+ Nalm6 tumors (Fig. 5a). As a positive control, we generated CD19-specific CARTs from a healthy donor using both CD3/28 beads + IL-7 and IL-15 and CDNPs. While the selected dose was non-therapeutic, we observed superior anti-tumor function of CDNP-activated T cells regardless of donor source, including from two CLL patients as well as a healthy donor (Fig. 5b–e). Notably, CDNP-activated CARTs from one patient led to significantly prolonged survival relative to mice who received CARTs from a healthy donor (Fig. 5f). Together, this data shows the capacity of CDNPs to manufacture products that failed CD3/28 coated bead manufacturing into highly active therapeutic products.

### cGMP CDNPs generate CARTs with potent activity in patients.

**Figure 6:**
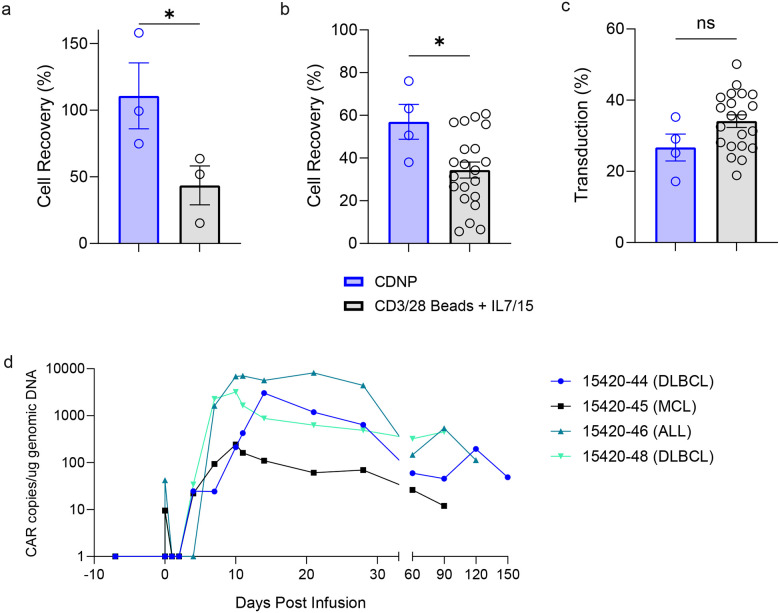
cGMP CDNPs generate CARTs with potent activity in patients. Patient T cells for preclinical expansion studies (n=3) (**a**) or T cells from patients enrolled in NCT04684563 (**b**, **c**) were activated by CDNPs (**a**) cGMP-grade CDNPs (**b**) (n=4) or cGMP CD3/28 beads + IL-7 and IL-15 (n=21) and transduced with a clinical grade lentiviral vector co-expressing 19BBz CAR and hIL-18 (19BBz-T2A-hIL18) on Day 0 (CDNP) or Day 1 (Beads), cultured for 3 days, and the percentage of T cells recovered after harvest is shown. **c**, Summary transduction efficiency of patient T cell products. Each dot represents a unique patient T cell manufacturing run. **d**, CART persistence as measured by CAR genomic copies in peripheral blood was performed in triplicate using qPCR. **a**, Two-sided t test was used in pairwise comparisons between two groups. **b**,**c**, Mann-Whitney test was used in unpaired, nonparametric comparisons between two groups.

In a recently described 3-day, bead-based manufacturing protocol of IL-18 armored, CD19-directed CARTs, one out of 22 eligible patients had a documented manufacturing failure with this novel process and 8 out of 21 manufactured products did not meet the assigned target dose^69^. As part of this ongoing Phase I clinical trial testing CD19-directed CARTs armored with IL-18^69^, we sought to determine the ability of CDNPs + IL-7 and IL-15 to facilitate three day manufacturing of these CARTs. To do this, a K.a3.86.BBL.m7.15 aAPC master cell bank was generated in accordance with good manufacturing practices and underwent FDA mandated testing for identity, sterility, and adventitious agents. With this master cell bank, CDNPs were manufactured under cGMP conditions. Consistent with our findings in the preclinical setting (Fig. 6a), we observed a significant improvement in cell recovery when cGMP-grade CDNPs were used to manufacture CARTs for infusion in the clinical setting after 3 days of expansion compared to that of CD3/28 beads + IL-7 and IL-15 (Fig. 6b). Incorporating Day 0 transduction in the manufacturing process also allowed CDNP-activated CARTs to maintain robust transduction efficiency with less manipulation of the product (Fig. 6c). We next assessed the safety, feasibility, and preliminary efficacy of the CDNP-generated anti-CD19 CARTs secreting IL-18 in patients with CD19+ B-cell neoplasms who experienced prior failure of an FDA-approved CART product. Following standard lymphodepleting chemotherapy, we administered 7 million CDNP-generated CAR+ T cells which was the recommended dose for expansion based on the dose-finding phase of the study with CD3/28 bead-generated CARTs^69^. Six individuals were screened, of whom 5 were eligible and enrolled in the study including 4 with non-Hodgkin lymphoma and 1 with ALL (see **Consort Diagram** Supplementary Fig. 3). One individual’s product became contaminated during the manufacturing process and was considered a manufacturing failure, while 4 individuals received their product (see Supplementary Table 1,2 **for a summary of patient demographics**). Adverse event monitoring revealed a similar favorable profile as to what was reported with CD3/28-bead manufactured huCART19-IL18 cells (Supplementary Table 3) with two patients having mild (grade 1) cytokine release syndrome, one with transient neurotoxicity (grade 3) and two patients who had incomplete blood count recovery 3 months post-CART infusion (Supplementary Table 4,5). Complete responses (CR) were observed in 3 of the 4 individuals at 3 months (Table 1). Additionally, we observed high engraftment of CARTs, especially in patients with CR (Fig. 6d). While we do not have enough data to compare outcomes of CD19 CARTs manufactured by CDNPs are versus those manufactured using CD3/28 beads + IL-7 and IL-15, it is clear that CDNPs can produce T cells with potent therapeutic activity, as 7 million of these IL-18 producing CD19 CARTs were able to induce CRs in heavily pre-treated patients who had previously experienced failure of a currently FDA-approved CART products.

In summary, our data demonstrates that cell-derived nanoparticles retain the superior stimulatory capacity of engineered aAPC cell lines to expand functional CARTs of all T cell subsets examined and are highly compatible with accelerated manufacturing processes. Our humanized mouse data and use of CDNPs to manufacture T cells for a Phase I clinical trial demonstrate clinical proof of concept and collectively strengthen the rationale to use CDNP to manufacture T cells for adoptive T cell therapy.

## Discussion

The growing number of promising clinical trials and FDA approved therapies employing adoptively transferred T cells highlight the therapeutic potential of this approach^70–72^. However, manufacturing these T cells remains a complicated, labor-intensive, and expensive process which fails between 5–30% of the time to generate a personalized therapy^16–30^. CDNPs address these limitations and offer an improved platform by which T cells are manufactured for adoptive T cell therapy. Our studies focused on generating CD19 CARTs but CDNPs are a flexible platform by which costimulatory and cytokine signals can be easily added and removed, facilitating the ability to optimize a CDNP for any adoptive T cell therapy product. For example, Lifileucel is an FDA approved autologous tumor-infiltrating lymphocyte (TIL) product manufactured from a patient’s resected tumor that uses a 22-day manufacturing process to treat advanced melanoma^73,74^. The number of CD39+CD103+PD-1- CD8+ T-cell population within the tumor is associated with improved prognosis in melanoma^75^. aAPCs could be designed that specifically enrich this population and this may lead to a more streamlined and shorter manufacturing process and improved outcomes of this therapy. Likewise, both natural and engineered Tregs are being explored as therapeutics against GVHD and autoimmune disease^76,77^. Tregs are hypoproliferative *ex vivo* and a rare T cell population that must be isolated prior to manufacturing, necessitating complicated and long manufacturing processes. aAPCs designed to optimally expand Tregs significantly reduced manufacturing times^49–51^ and resulted in a product that successfully mitigated GVHD after patients received a bone marrow transplant^53^. Based on these findings, optimized CDNPs could be developed to expand Tregs that could also streamline the process and improve the potency of this cell therapy.

A striking finding of our study was that CDNPs were able to accelerate the time to first division enabling up to 40% of T cells to divide once after only 48hrs of activation. Curiously, the aAPC by which the CDNPs were derived did not have this ability, indicating that this difference was not the result of having additional signals such as 4–1BBL. Rather, this finding suggests that the physical nature of CDNPs being small membrane particles rather than intact cells were responsible for the ability of CDNPs to promote more rapid cell division. Gett and Hodges performed elegant studies in which they determined the time to first division could be altered by the strength of signal 1 and signal 2 whereas subsequent divisions were largely controlled by cytokines such as IL-2^78^. Other studies have shown that that heterogeneity by which T cells complete their first division is a function of how much active SHP-1 is present with cells having less SHP-1 entering the cell cycle faster^79^. Thus, CDNPs may provide a stronger signal 1 and 2 than CD3/28 coated beads and allow T cells with less SHP-1 to enter the cell cycle faster.

This more rapid proliferation enables a more streamlined and efficient T cell manufacturing process. Shorter manufacturing times are associated with better clinical outcomes, a lower therapeutic dose of infused engineered T cells, and less labor and reagents^31,69,80^. Moreover, the ability to transduce and activate at the same time and the lack of a requirement to remove CDNPs streamlines the manufacturing process, improves product recovery and consistency, and reduces the amount of hands-on manipulation required to generate a therapeutic product. We were able to demonstrate all this using cGMP CDNPs as part of Phase I clinical trial in which impressive clinical outcomes were obtained. This initial clinical success facilitates wider use of CDNPs to manufacture adoptive T cell therapies. Importantly, CDNPs were able to manufacture T cells that were recalcitrant to state-of-the-art manufacturing platforms. Prior therapy and age skew a patient T cell population to highly differentiated effector memory cells. There is much discussion on what type of T cell is best to cure a particular disease with most arguing that less differentiated T cells are preferred^81,82^. For CART therapy, patients that have a higher proportion of less differentiated T cells in their infusion product do have better clinical outcomes^83–86^. However, many patients are lacking these cells, and we would argue that all effector T cells can play role in disease eradication and a product that contains highly differentiated T cells is better than no product at all. Moreover, it is important to note that CDNPs did not further differentiate a less differentiated T cell into a more differentiated T cell *ex vivo*. Rather, our studies using purified central memory and effector memory T cells show that CDNPs grow both populations better than CD3/28 beads and these T cells maintain their starting phenotype during the manufacturing process. Thus, the ability of CDNPs to generate products with effector memory T cells reflects improved growth of effector memory rather than differentiation of central memory into effector memory. It will be interesting to see the clinical outcomes of individuals who received CART therapy as a result of their T cells being manufactured by CDNPs who otherwise would have failed to receive a product if their T cells had been manufactured with CD3/28 coated beads. Our humanized mouse data suggests that these T cells will be effective therapeutic agents. Moreover, our *in vivo* studies suggest that T cells manufactured using CDNPs are more potent than those manufactured using CD3/28 coated beads. However, even if CDNP manufactured T cells have the same potency as those manufactured with CD3/28 coated beads, the ability of CDNPs to manufacture a greater percentage of patients with less effort and cost justifies their widespread use.

## Materials and Methods

### Lentivirus production

Lentiviral particles were generated using packaging expression vectors that encode VSV or Cocal glycoprotein, HIV Rev and HIV Gag/Pol (pTRPE pVSV-g, pCocal-g, pTRPE.Rev and pTRPE g/p, respectively), and were synthesized using DNA 2.0 or ATUM. The pTRPE transfer vector contained a murine FMC63-based, second-generation CAR construct (CD8 hinge, 4– 1BB co-stimulatory domain, and CD3ζ signaling domain) as previously described^62^. The packaging plasmids along with the appropriate pTRPE transfer vector were transfected into HEK293T cells using Lipofectamine 2000 (Life Technologies). At 24 and 48 h after transfection, the HEK293T cell supernatant was collected, filtered through a 0.45-μm syringe-driven filter and then concentrated by ultracentrifugation for 2.5 h at 25,000 rpm. at 4°C. The supernatant was aspirated and the virus pellet was resuspended in 1000 μl of complete RPMI and stored at −80°C. GMP huCART-19-IL18 lentiviral vector was produced by UPenn GMP Core as previously described^69^.

### aAPC and cell line culture

All K562-based aAPCs, 293T cells and NALM6 cells were grown in RPMI medium (Gibco) supplemented with 10% Fetal Bovine Serum (FBS), 1x GlutaMAX (Gibco), 10 mM HEPES (Gibco), and 1x Penicillin-Streptomycin (Gibco) at 37°C with 5% CO2. aAPCs were maintained at 25k-50k cells/mL every 2–3 days. Leukemic cell line (Nalm6.CBG.GFP) was previously described^87^. To generate our aAPCs, we dual transduced parental K562 cells with lentiviruses encoding an scFv against human CD3ε fused to the CD8α hinge and transmembrane domains, full length human CD86, full length human 4–1BBL, full length human IL-7 fused to the IgG4 hinge and constant regions and CD4 transmembrane domain, full length human IL-15Rα, and full length human IL-15. Cells were single cell sorted on a FACS Influx, Aria, or Symphony cell sorter (BD) into a 96-well round bottom plate containing complete RPMI and 100 mg/mL Normocin (Invivogen). After 3 weeks of incubation at 37°C with 5% CO2, individual clonal populations were screened for transgene surface expression and T cell stimulatory function. Clones with high and stable transgene surface expression and stimulatory function was then further expanded, γ-irradiated (100 gy), and frozen for future use or nitrogen cavitation to produce CDNPs.

### CDNP Generation

Engineered K562 cells were first expanded for 10–13 days in a G-Rex gas-permeable cell culture device (Wilson-Wolf) and harvested using the GatheRex closed system (Wilson-Wolf). Cells were then washed and formulated into cavitation buffer using the LOVO system (Fresenius Kabi) at a final concentration of 2.5 × 10^7^ cells/mL. The cavitation buffer consisted of 50 mM HEPES, 150 mM NaCl, and 2 mM MgCl_2_ in sterile water. Prior to cell disruption by cavitation, the cell suspension was spiked with protease inhibitors (1:50 dilution, MilliporeSigma) and endonuclease (1000 U/mL, MilliporeSigma). Cavitation was performed using two consecutive incubations at 400 psi with ultra-high purity nitrogen gas for 30 minutes at 4 °C (Parr Instruments). After cavitation, the crude product was rotated for 1 hour at room temperature and then frozen at −80 °C for further refinement. For refinement, the crude product was thawed and spiked with an additional dose of endonuclease (1000 U/mL, MilliporeSigma) before undergoing a first wash with the LOVO system (Fresenius Kabi) to remove larger particles. A second filtration step was subsequently performed to remove smaller particles, concentrate the product, and diafilter it into OpTmizer media (Thermo Fisher Scientific) using tangential flow filtration (TFF, Repligen). The refined product was then aliquoted and stored at −80 °C for further characterization and use.

### Human T cell isolation, transduction, and expansion

De-identified human donor T cells were purified by the University of Pennsylvania Human Immunology Core (RRID: SCR_022380). T cells were placed in culture at 10^6^ cells per mL in complete CTS OpTmizer T-Cell Expansion SFM (Gibco) with 1% Penicillin-Streptomycin, 2 mM GlutaMax and 25 mM HEPES buffer. T cell expansion medium was complemented with human IL-15 (10 ng/mL, Biolegend) and IL-7 (10 ng/mL, R&D). T cells were stimulated at a 3:1 (bead/cell) ratio with anti-CD3/CD28 Dynabeads (Life Technologies), a 1:2 (K562/T cell) ratio with irradiated K562 (100 gy) expressing various stimulatory molecules, or an optimal amount of cell-derived nanoparticles, and incubated at 37°C, 5% CO2 and 95% humidity. 15 min before CDNP stimulation or after 24 h of initial stimulation by Dynabeads or Irradiated K562, lentivirus for CAR transduction was added at a multiplicity of infection (MOI) of 5. 5 days after T cell activation, magnetic separation was used to remove Dynabeads. Culture media was added every 1 to 2 days to feed cells and adjust cell counts to 0.5 × 10^6^ cells per mL.

### Flow cytometry

Washed cells were resuspended in 100 μl of PBS containing 2 mM EDTA and 2% FBS, and then surface stained with anti-human antibodies from BioLegend: CD3-BV605 (OKT3), CD4-BV421/BV785 (Clone: OKT4), CD8α-BV510 (RPA-T8), CD27-BV650 (LG.3A10), CCR7 PE/APC (G043H7); BD Biosciences: CD45RA-BV421 (HI100), CD45RO-BUV737 (UCHL1), CD62L-BUV395 (SK11); Jackson Immunoresearch: Goat a-Mouse IgG F(ab’)2-AF488; R&D: Rabbit anti-G4SL mAb-AF647 (E702V); ACROBiosystems: CD19 protein-AF647/FITC; or Fixable Viability Dye eFluor 780 (eBioscience). All extracellular stained cells were fixed in 2% paraformaldehyde before analysis. Flow cytometry data were acquired on BD LSRFortessa instrument using BD FACSDiva Software v.8.0.1 (BD Biosciences). Data were analyzed using FlowJo software with positive populations defined using fluorescence minus one (FMO) gating or fully stained negative samples.

### T cell sorting

De-identified human T cells were obtained from the Human Immunology Core at the University of Pennsylvania under an institutional review board (IRB)-approved protocol and stained using antibodies for CD4-BV421 (Clone: OKT4), CD8α-BV785 (RPA-T8), CD45RA-PE (HI100), CD45RO-BUV737 (UCHL1), CD27-BV650 (LG.3A10), CCR7- APC (G043H7). T_CM_ (CD45RA-CCR7+CD27+), and T_EM_ cells (CD45RA-CCR7−CD27−) were sorted to high purity using a BD FACS Aria or Symphony.

### Intracellular cytokine staining

Transduced T cells were co-cultured with target cells at a 1:2 ratio or with 3 mg/mL PMA (Sigma Aldrich) and 1 mg/mL Ionomycin (Sigma Aldrich) for 5 h in the presence of GolgiPlug Protein Transport Inhibitor (BD Biosciences). Cells were then stained for surface markers, fixed with Fixation Medium A (Thermo Fisher Scientific), washed, and stained with IL2-BV605 (MQ1–17H2, BD), TNFa-BUV395 (MAB11, BD), and IFNg-BUV737 (4S.B3, BD) antibodies in Fixation Medium B (Thermo Fisher Scientific) for 20 min. Following a final wash, cells were analyzed on a BD Fortessa flow cytometer.

### Humanized mice

All animal studies were performed under an approved Institutional Animal Care and Use Committee protocol at the University of Pennsylvania (807158). 6–8-week-old male NOD/SCID/IL2Rγ−/− (NSG) mice obtained from the Jackson Laboratory (RRID: IMSR_JAX:005557) were purchased from the Stem Cell and Xenograft Core of the University of Pennsylvania (RRID:SCR_010035). Mice were maintained in pathogen-free facilities at the University of Pennsylvania. Microisolator cages were used to house mice, and mice were fed autoclaved food and water. Animal rooms were maintained at 72 ± 2°F (22.2 ± 1.1 °C) and 30–70% relative humidity and were on a 12:12-h light/dark cycle. NSG mice were injected intravenously (i.v.) with 0.75 × 10^6^ Nalm6.CBG.GFP cells line on day 0. On day 6 tumor engraftment was measured by bioluminescent imaging (BLI) and study groups were made after distributing the mice equally into treatment groups based on BLI values. On day 5, CARTs were injected in respective mice groups in 100 μl of PBS at the indicated concentration. Imaging was performed every week following injections to establish the kinetics of tumor burden and eradication by CARTs. Bioluminescent imaging was performed by using an IVIS^®^ Lumina III In Vivo Imaging System (PerkinElmer; RRID: SCR_025239) and analyzed with Living Image software v. 4.3.1 (PerkinElmer). Animals were euthanized at the end of the experiment according to the IACUC protocols.

#### Generation of Master Cell Bank:

The Product Development Laboratory (PDL) at the Center for Cellular Immunotherapies (CCI) developed and executed a large-scale, clinical-grade manufacturing process to establish engineered-K562 cell banks for use as feeder material in cell-derived nanoparticle (CDNP) production. An initial full-scale manufacturing run was performed to establish a Master Cell Bank (MCB) derived from authenticated, media-adapted engineered-K562 cells. Cells were expanded for up to 13 days in a G-Rex gas-permeable cell culture device (Wilson Wolf) and harvested using the GatheRex closed system (Wilson Wolf), yielding up to 1.8 × 10^10^ total cells per run. Harvested cells were washed and formulated into cryopreservation medium, [1:1 volume of Cryostore CS10 and Plasmalyte] at multiple cell concentrations to support future clinical manufacturing, quality control testing, and release test. A second manufacturing run was subsequently performed to expand the MCB into a Working Cell Bank (WCB) using the same validated process. The master cell and working cell banks were generated in accordance with the FDA document “Points to Consider in the Characterization of Cell Lines Used to Produce Biologicals (1993)”.

#### cGMP manufacturing IL-18 armored CARTs.

CART cells were manufactured by the Clinical Cell and Vaccine Production Facility (CVPF) at the University of Pennsylvania. Briefly, autologous peripheral blood lymphocytes were collected via leukapheresis, washed and cryopreserved until manufacturing initiation. On the first day of manufacturing (Day 0), the leukopacks were thawed and washed using a LOVO closed system. The CD4 and CD8 T cells were enriched by labeling with CD4 and CD8 magnetic beads (Miltenyi Biotech) followed by magnetic separation in a CliniMACS device. The enriched T cells were seeded in OpTmizer based media supplemented with CTS T-Cell Supplement, GlutaMAX (2mM), Human Serum (5%), IL7 and IL15 (5 ng/mL each). The cultures were transduced with the GMP huCART-19-IL18 lentiviral vector and then activated using the GMP CDNPs at 1:10,000 T:CDNP ratio. The cultures were placed in incubator until day 3 when they were harvested. On the harvest day, cells were washed, samples were collected for release testing, and the products were formulated in cryomedia (CS10, BioLife Solutions) at the infusion target dose. The formulated products were cryopreserved using a controlled-rate freezer and stored in a monitored freezer in vapor phase liquid nitrogen. The products were released upon completing and passing all pre-established release testing. We determined the degree of huCART19-IL18 expansion and persistence by measuring the number of copies of huCART19 transgene per microgram of genomic DNA using real-time quantitative polymerase-chain-reaction (qPCR) assays as previously described^69^.

### Clinical Trial

Patients receiving CNDP were treated on a phase 1 trial of huCART19-IL18 for patients with CD19+ B-cell malignancies at the University of Pennsylvania (NCT04684563). Cohort D was established to evaluate feasibility and safety of CDNPs in manufacturing. All the patients had previous failure of CD19-directed CAR T-cell therapy. Patients received a flat dose of 7 million huCART19-IL18 cells. The trial was approved by the institutional review board at the University of Pennsylvania, conducted in accordance with the principles of the Declaration of Helsinki, and overseen by an independent data and safety monitoring board.

### Statistical analysis

All statistical analyses were performed using GraphPad Prism. Two-sided t test was used in unpaired or pairwise comparisons of two groups of normal or log-transformed data. Two-sided ratio t test was used in pairwise log-normal comparisons of two groups. Two-sided Wilcoxon matched-pairs signed-rank test was used in nonparametric pairwise comparisons of two groups. Mann-Whitney test was used in nonparametric, unpaired comparisons between two groups. Repeated measures one-way analysis of variance (ANOVA) with Tukey’s or Bennett’s post hoc test was used for parametric, pairwise comparisons among three or more groups of one factor. Repeated measures one-way ANOVA with Holm-Sidak’s post hoc test was used for parametric, pairwise, log-transformed comparisons among three or more groups of one factor. Friedman’s test followed by Dunn’s multiple comparisons test was used for nonparametric pairwise comparisons among three or more groups of one factor. Kaplan-Meier survival data were analyzed using a log-rank (Mantel-Cox) test. Bar and line graphs were generated using GraphPad Prism or RStudio. All data are generated from or are representative of at least three donors and expressed as means ± SEM. P values of less than 0.05 were considered significant. Additional statistical details of experiments and sample sizes can be found in figure legends.

## Supplementary Material

1

## Figures and Tables

**Table 1 |:** Summary of patient characteristics, safety events, and treatment responses

Subject ID	Disease Indication	Notable Safety Events	Prior Treatment History	Total CART Dose Administered	Preliminary Response
**15420-44**	DLBCL NOS	No	**# Prior Lines:** 5 **Prior CART:** 2024	7×10^6^ cells	Complete Metabolic Response
**15420-45**	MCL	No	**# Prior Lines:** 7 **Prior CART:** 2024	7×10^6^ cells	Progress Disease
**15420-46**	ALL	Gr 3 CAR Neurotoxicity	**# Prior Lines:** 10 **Prior CART:** 2022	7×10^6^ cells	Complete Response with Incomplete Blood Count Recovery
**15420-48**	DLBCL NOS	Gr 1 CRS	**# Prior Lines:** 6 **Prior CART:** 2022	7×10^6^ cells	Complete Metabolic Response

## Data Availability

All data generated and/or analyzed during the current study are available from the corresponding author upon reasonable request.
