# Supplementary material for: Cell-derived Nanoparticles Provide a Robust Platform to Manufacture Therapeutic T cells
Source: Res Sq. 2026 Feb 4:rs.3.rs-8436008. Preprint. [Version 1] doi: 10.21203/rs.3.rs-8436008/v1 (PMC12889848; doi:10.21203/rs.3.rs-8436008/v1)
Supplement: 1 [file NIHPPrs8436008V1-supplement-1.pdf]

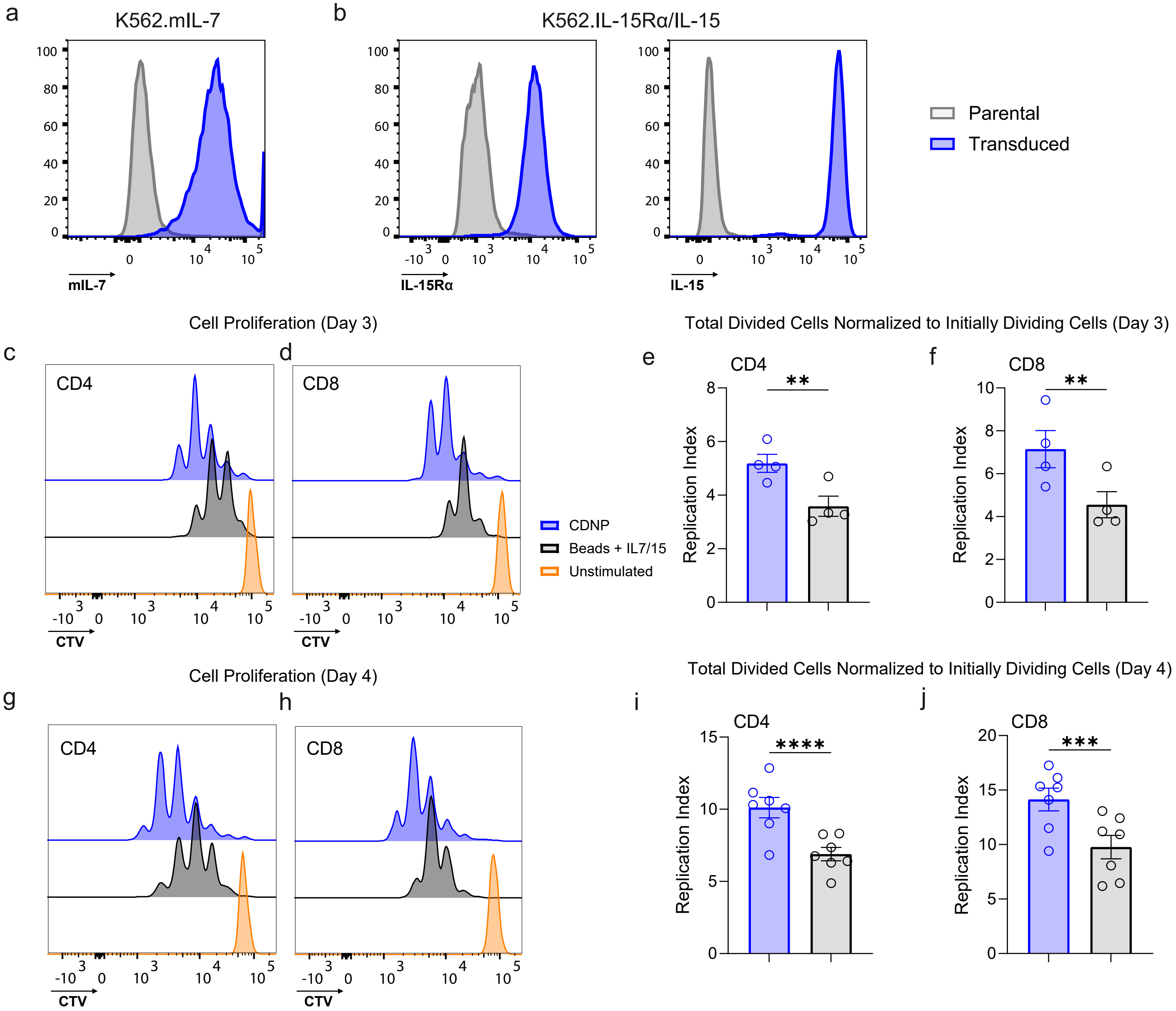

Deng et al, Supplementary Figure 1.

Supplementary Figure 1: Cavitation of aAPCs generates CDNPs that retain costimulatory and cytokine activity and enable simultaneous transduction and activation of CARTs. a,**b**, K562 cells were transduced with mIL-7 (**a**), or IL-15Rα/IL-15 (**b**), with surface gene expression evaluated via flow cytometry; CDNPs were subsequently produced from K562 cells expressing combinations of cytokines. **c**-**j**, Primary human T cells were labeled with CTV and activated by soluble IL-7 or IL-15, CD3/28 beads, or CDNPs presenting combinations of cytokines. **c**-**f**, Primary human CD4 (**c**,**e**) and CD8 (**d**,**f**) T cells were expanded for 3 days. Summary data for 4 independent experiments is shown in **e** and **f** (n=4). **g**-**j**, Primary human CD4 (**g**,**i**) and CD8 (**h**,**j**) T cells were expanded for 4 days. Summary data for 7 independent experiments is shown in **i** and **j** (n=7). Replication index is calculated as the total number of divided cells normalized to the number of initially dividing cells. All data are from at least three independent donors and expressed as mean ± SEM. All statistical analysis was performed using two-sided ratio t test for pairwise log-normal comparisons. The same color schemes apply to figures **a** and **b**, and the same color schemes apply to figures **c**-**j**.

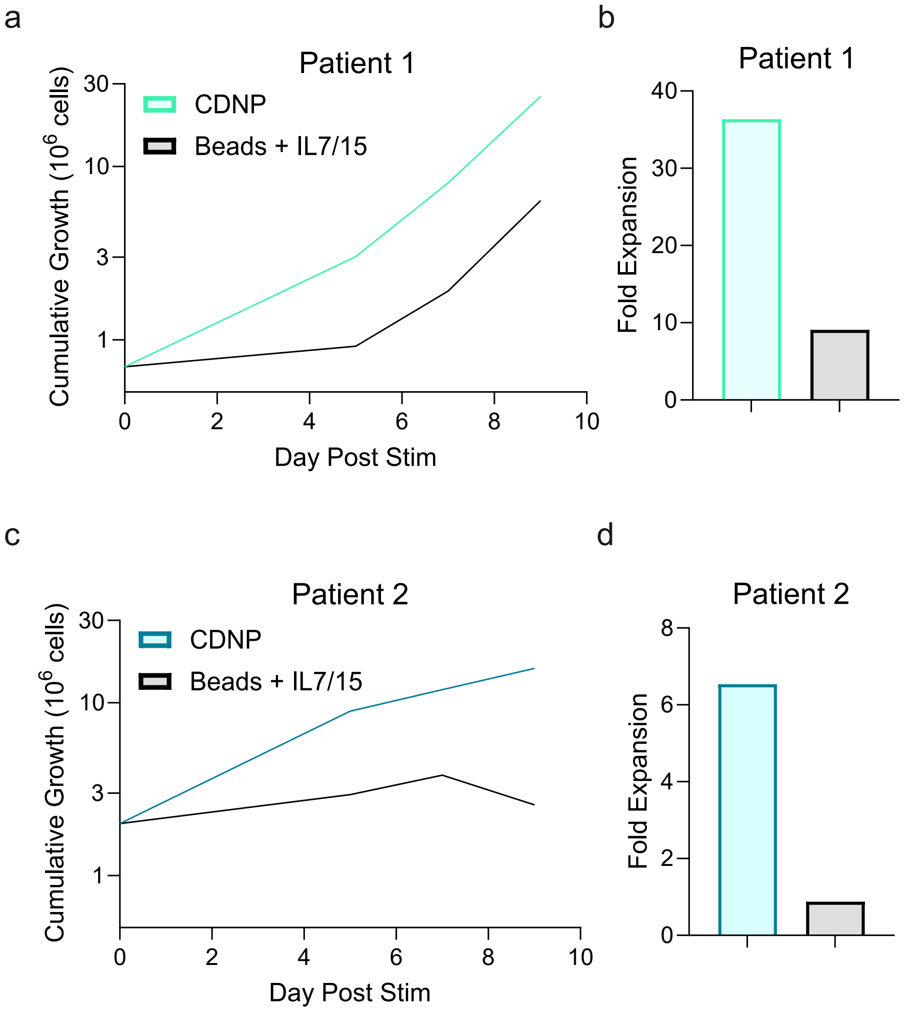

Deng et al, Supplementary Figure 2.

Supplementary Figure 2: CDNP-stimulated CARTs from patient T cells that failed conventional manufacturing maintain robust anti-tumor activity and prolong survival in vivo. a,**d**, T cells from two CLL patients were activated by CDNPs or CD3/28 beads + IL-7 and IL-15, transduced with 19BBz CAR on Day 0 (CDNP) or Day 1 (Beads), and expanded over a 9-day culture period. Growth (**a**) and fold expansion (**b**) of 19BBz CARTs were measured for Patient 1. Growth (**c**) and fold expansion (**d**) of 19BBz CARTs were measured for Patient 2.

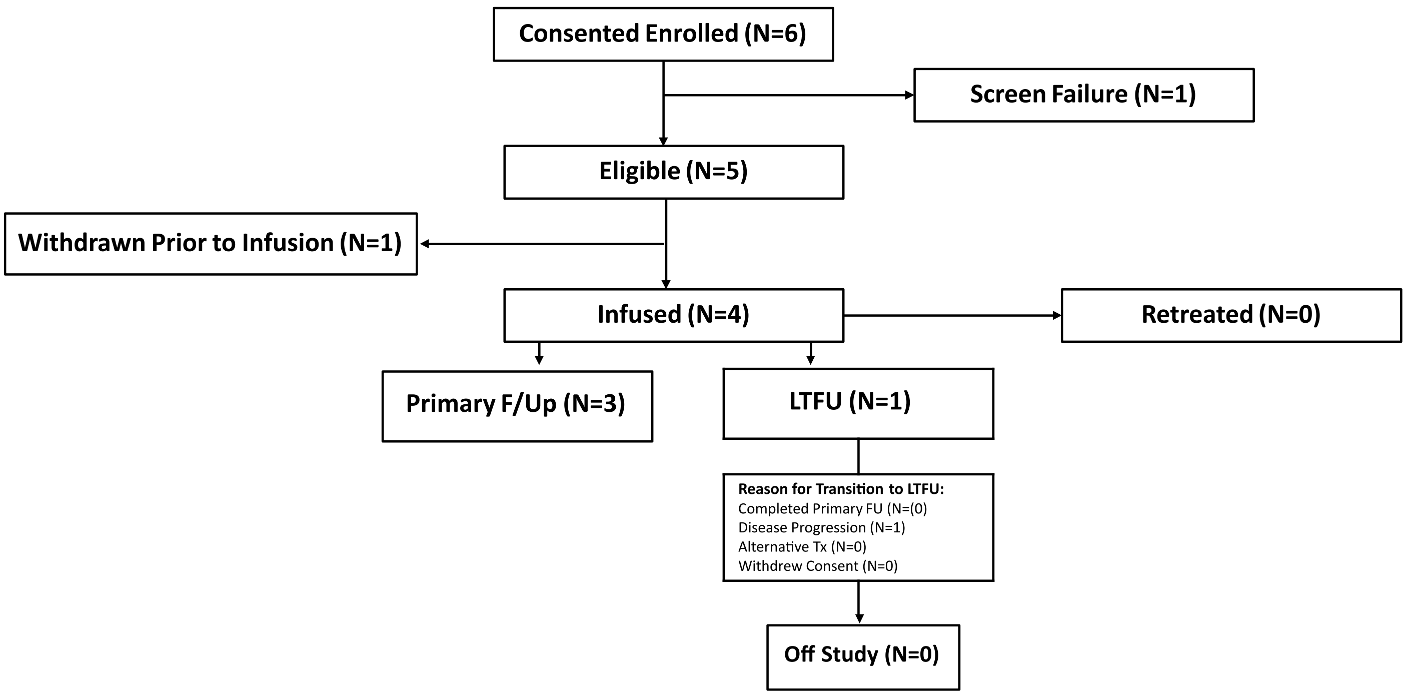

Data cutoff date: 11/30/2025

Deng et al, Supplementary Figure 3.

Supplementary Figure 3: CONSORT diagram detailing participants consented and screened.

Supplementary Table 1 | Patient demographics

Cohort (NHL/ALL)				
**Status**	**Total Screened N=6**	**Screen Failed N=1**	**Eligible N=5**	**Infused N=4**
**Legal Sex**				
**Male**	2 (33.3%)	1 (100.0%)	1 (20.0%)	1 (25.0%)
**Female**	4 (66.7%)	0 (0.0%)	4 (80.0%)	3 (75.0%)
**Nonbinary (X)**	0 (0.0%)	0 (0.0%)	0 (0.0%)	0 (0.0%)
**Not Reported**	0 (0.0%)	0 (0.0%)	0 (0.0%)	0 (0.0%)
**Age at Consent**				
**Mean SD**	52.33 (18.20)	33.00 (0.00)	56.20 (17.37)	53.50 (18.81)
**Median**	50.5	33	62	50.5
**Range**	33.00 - 76.00	33.00 - 33.00	37.00 - 76.00	37.00 - 76.00
**Race**				
**African American**	0 (0.0%)	0 (0.0%)	0 (0.0%)	0 (0.0%)
**Alaska Native**	0 (0.0%)	0 (0.0%)	0 (0.0%)	0 (0.0%)
**American Indian**	0 (0.0%)	0 (0.0%)	0 (0.0%)	0 (0.0%)
**Asian**	0 (0.0%)	0 (0.0%)	0 (0.0%)	0 (0.0%)
**Caucasian**	6 (100.0%)	1 (100.0%)	5 (100.0%)	4 (100.0%)
**Multiple Races**	0 (0.0%)	0 (0.0%)	0 (0.0%)	0 (0.0%)
**Pacific Islander**	0 (0.0%)	0 (0.0%)	0 (0.0%)	0 (0.0%)
**Other**	0 (0.0%)	0 (0.0%)	0 (0.0%)	0 (0.0%)
**Unknown**	0 (0.0%)	0 (0.0%)	0 (0.0%)	0 (0.0%)

Supplementary Table 2 | Infusion Details

Subject ID	Disease	Lymphodepleting Chemotherapy Regimen	Date of huCART19-IL18 Infusion	Cells Infused			
				Target Cell Dose	Total CART Cell Dose Administered	Total Cell Dose Administered	Met Target Dose
15420-44	Diffuse Large B-cell Lymphoma not otherwise specified (DLBCL NOS)	Bendamustine	06/23/25	$7x10^6$	$7x10^6$	$2.41x10^7$	Y
15420-45	Mantle cell lymphoma	Bendamustine	07/28/25	$7x10^6$	$7x10^6$	$4.07x10^7$	Y
15420-46	B-Cell Acute Lymphoblastic Leukemia	Fludarabine + Cyclophosphamide	07/31/25	$7x10^6$	$7x10^6$	$1.98x10^7$	Y
15420-48	Diffuse Large B-cell Lymphoma not otherwise specified (DLBCL NOS)	Bendamustine	08/25/25	$7x10^6$	$7x10^6$	$2.78x10^7$	Y

Supplementary Table 3 | Adverse events

Category<br>*Toxicity*	Grades				Total
	1	2	3	4	
**Blood and lymphatic system disorders**	**0**	**0**	**1**	**0**	**1**
Anemia	0	0	1	0	1
**Eye disorders**	**1**	**0**	**0**	**0**	**1**
Eye disorders - Other (Visual change)	1	0	0	0	1
**Gastrointestinal disorders**	**3**	**4**	**0**	**0**	**7**
Constipation	1	0	0	0	1
Diarrhea	0	2	0	0	2
Gastroesophageal reflux disease	0	1	0	0	1
Nausea	1	1	0	0	2
Vomiting	1	0	0	0	1
**General disorders and administration site conditions**	**1**	**0**	**0**	**0**	**1**
Fatigue	1	0	0	0	1
**Immune system disorders**	**2**	**0**	**0**	**0**	**2**
Cytokine release syndrome	2	0	0	0	2
**Infections and infestations**	**0**	**1**	**1**	**0**	**2**
Sepsis	0	0	1	0	1
Urinary tract infection	0	1	0	0	1
**Investigations**	**8**	**4**	**3**	**2**	**17**
Alanine aminotransferase increased	3	0	0	0	3
Aspartate aminotransferase increased	4	0	0	0	4
Lymphocyte count decreased	0	0	0	2	2
Neutrophil count decreased	0	2	1	0	3
Platelet count decreased	1	1	0	0	2
White blood cell decreased	0	1	2	0	3
**Metabolism and nutrition disorders**	**2**	**0**	**0**	**0**	**2**
Anorexia	1	0	0	0	1
Hypomagnesemia	1	0	0	0	1
**Musculoskeletal and connective tissue disorders**	**0**	**1**	**0**	**0**	**1**
Pain in extremity	0	1	0	0	1
**Nervous system disorders**	**0**	**0**	**1**	**0**	**1**
Nervous system disorders - Other (CAR neurotoxicity)	0	0	1	0	1
**Respiratory, thoracic and mediastinal disorders**	**1**	**0**	**0**	**0**	**1**
Epistaxis	1	0	0	0	1
**Skin and subcutaneous tissue disorders**	**1**	**2**	**0**	**0**	**3**
Alopecia	1	0	0	0	1
Skin and subcutaneous tissue disorders - Other (Rash)	0	1	0	0	1
Skin ulceration	0	1	0	0	1
**Vascular disorders**	**0**	**1**	**0**	**0**	**1**
Thromboembolic event	0	1	0	0	1
**Total**	**19**	**13**	**6**	**2**	**40**

Supplementary Table 4 | Response listing (NHL patients)

Subject ID	Current Response			Best Response/Timepoint[1]		Overall Response/Month 3		AEs (Y/N)	SAEs (Y/N)	Study Status	Reason for LTFU
	PET-Based	CT-Based	Timepoint	PET-Based	CT-Based	PET-Based	CT-Based				
15420-44	CMR	PR	Month 3	CMR/Month 3	PR/Month 3	CMR	PR	Y	N	Primary	
15420-45	PMD	PD	Month 3	PMD/Month 3	PD/Month 3	PMD	PD	Y	N	LTFU	Disease Progression
15420-48	CMR	CR	Month 3	CMR/Month 3	CR/Month 3	CMR	CR	Y	N	Primary	

[1] Best Response Timepoint reported as when first observed.

CMR – Complete Metabolic Response; CR – Complete Radiologic Response; PMR – Partial Metabolic Response; PR – Partial Response; SD – Stable Disease; PMD – Progressive Metabolic Disease; PD – Progressive Disease; NA – Not Assessed; Pending – Subjects who have not had at least one post-infusion response evaluation performed or have not yet reached the Month 3 study timepoint.

Supplementary Table 5 | Response listing (ALL patient)

Subject ID	Current Response			Best Response/Timepoint[1]		Overall Response/Day 28		AEs (Y/N)	SAEs (Y/N)	Study Status	Reason for LTFU
	Overall Response	Extramedullary Disease w/o Bone Marrow Involvement	Timepoint	Overall Response	Extramedullary Disease w/o Bone Marrow Involvement	Overall Response	Extramedullary Disease w/o Bone Marrow Involvement				
**15420-46**	CRi	SD	Month 3	CRi/Month 3	SD/Month 3	NA	NA	Y	Y	Primary	

[1] Best Response Timepoint reported as when first observed.

CR – Complete Remission; CRi – Complete Remission with Incomplete Marrow Recovery; CRu – Complete Remission with Residual Mediastinal Disease; TF – Treatment Failure; RD – Relapsed Disease; PR – Partial Remission; SD – Stable Disease; PD – Progressive Disease; NA – Not Assessed; Pending – Subjects who have not had at least one post-infusion response evaluation performed or have not yet reached the Day 28 study timepoint.
